# Comparative Analysis of Habitat Expansion Mechanisms for Four Invasive *Amaranthaceae* Plants Under Current and Future Climates Using MaxEnt

**DOI:** 10.3390/plants14152363

**Published:** 2025-08-01

**Authors:** Mao Lin, Xingzhuang Ye, Zixin Zhao, Shipin Chen, Bao Liu

**Affiliations:** College of Forestry, Fujian Agriculture and Forestry University, Fuzhou 350002, China; 13408543373@163.com (M.L.); yxz@fafu.edu.cn (X.Y.); 15131902213@163.com (Z.Z.); fjcsp@126.com (S.C.)

**Keywords:** climate change, MaxEnt model, *Amaranthaceae*, habitat suitability, invasive plants, bioclimatic variables

## Abstract

As China’s first systematic assessment of high-risk *Amaranthaceae* invaders, this study addresses a critical knowledge gap identified in the National Invasive Species Inventory, in which four invasive *Amaranthaceae* species (*Dysphania ambrosioides*, *Celosia argentea*, *Amaranthus palmeri*, and *Amaranthus spinosus*) are prioritized due to CNY 2.6 billion annual ecosystem damages in China. By coupling multi-species comparative analysis with a parameter-optimized Maximum Entropy (MaxEnt) model integrating climate, soil, and topographical variables in China under Shared Socioeconomic Pathways (SSP) 126/245/585 scenarios, we reveal divergent expansion mechanisms (e.g., 247 km faster northward shift in *A. palmeri* than *D. ambrosioides*) that redefine invasion corridors in the North China Plain. Under current conditions, the suitable habitats of these species span from 92° E to 129° E and 18° N to 49° N, with high-risk zones concentrated in central and southern China, including the Yunnan–Guizhou–Sichuan region and the North China Plain. Temperature variables (Bio: Bioclimatic Variables; Bio6, Bio11) were the primary contributors based on permutation importance (e.g., Bio11 explained 56.4% for *C. argentea*), while altitude (e.g., 27.3% for *A. palmeri*) and UV-B (e.g., 16.2% for *A. palmeri*) exerted lower influence. Model validation confirmed high accuracy (mean area under the curve (AUC) > 0.86 and true skill statistic (TSS) > 0.6). By the 2090s, all species showed net habitat expansion overall, although *D. ambrosioides* exhibited net total contractions during mid-century under the SSP126/245 scenarios, *C. argentea* experienced reduced total suitability during the 2050s–2070s despite high-suitability growth, and *A. palmeri* and *A. spinosus* expanded significantly in both total and highly suitable habitat. All species shifted their distribution centroids northward, aligning with warming trends. Overall, these findings highlight the critical role of temperature in driving range dynamics and underscore the need for latitude-specific monitoring strategies to mitigate invasion risks, providing a scientific basis for adaptive management under global climate change.

## 1. Introduction

Global biodiversity faces dual crises: climate-driven habitat shifts and accelerating biological invasions [1]. Over the past 100 years, anthropogenic greenhouse gas emissions have elevated Earth’s surface temperature by 1.2 °C [2], with projections indicating a 1.5 °C threshold breach by 2040 under current trajectories [3]. Invasive species, as a term, refers to plants sourced from outside the local ecosystem [4], and they pose a serious threat to ecological security [5]. The increase in biological invasions will have serious consequences for the country’s economy and local livelihoods [6]. Concurrently, invasive species facilitated by intensified climate change have caused annual economic losses exceeding USD 160 billion worldwide [7] by 2017. These threats converge critically in China, where the number of invasive species has increased by 277 species between 2005 and 2020 [8], predominantly through agricultural imports [9]. Numerous alien plants have invaded China through globalization [10] because of their rapid growth rates and exceptional survival capacities [11], and climate change may further influence invasion dynamics by altering establishment and dispersal processes [12]. Consequently, in the context of climate change and extreme weather events, accurate prediction of invasive plant distributions and establishment of early warning systems are critical for developing effective containment strategies.

In recent years, the Ecological Niche Model (ENM) has emerged as a pivotal tool for forecasting the potential distribution and habitat suitability of invasive species [13], including CLIMEX [14], GARP [15], and MaxEnt [16]. Among these, the MaxEnt model is widely adopted for invasive species due to its robust performance with incomplete datasets and small sample sizes [17], though some scholars argue that it may overestimate habitat suitability when anthropogenic dispersal vectors decouple distributions from climatic constraints. Previous studies have identified ecologically sensitive areas by MaxEnt for invasive plants such as *Erigeron canadensis* [18], *Phragmites australis* [19], and *Ulex europaeus* [20], while integrated approaches combining MaxEnt with RK have been applied to predict distributions of *Ambrosia artemisiifolia* and *Ambrosia trifida* [21]. While MaxEnt applications are common, no study has quantified niche plasticity gradients among co-invading Amaranthaceae under anthropogenic drivers—a key gap for China’s biosecurity.

The *Amaranthaceae* family comprises approximately 70 genera and 900 species worldwide, with 15 genera (one introduced) and 44 species (three endemic, 14 introduced) in China [22]. Its invasive members, including *D. ambrosioides* and *A. palmeri*, combine high fecundity, stress tolerance, and C4 photosynthetic efficiency—traits poised to exploit warming climates [23]. *D. ambrosioides*, native to Mexico and Latin America, is now widespread across Europe, Africa, and Asia [24]. *A. palmeri*, indigenous to the southwestern U.S. and Mexican deserts and recently invasive in South America, Asia, Europe, Argentina, and Brazil [25], was first reported in Beijing (1985), with subsequent nationwide spread [26]. The successful establishment of invasive plants is influenced by species traits, climate, and soil factors. Although these species have colonized diverse regions, from Beijing to Yunnan, previous studies focused on their chemical traits, medicinal value [27], and allelopathic effects [28], but their synergistic response to warming climates necessitates urgent quantification [29].

**Figure 1:**
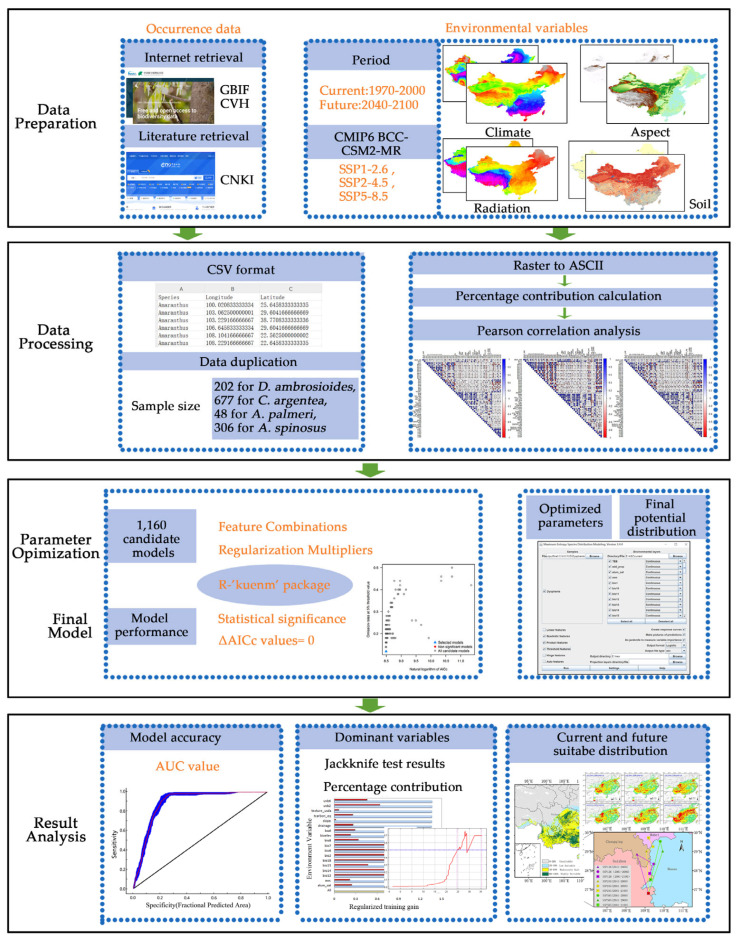
The analytical workflow diagram.

In this study, we address these gaps by coupling MaxEnt modeling with ArcGIS to analyze four high-risk *Amaranthaceae* invaders (*D. ambrosioides*, *C. argentea*, *A. palmeri*, and *A. spinosus*). All four species are listed among China’s highest-risk invasive aliens in the updated national inventory [8], with documented impacts on ecosystems exceeding CNY 2.6 billion annually. Based on the actual distribution data and relevant environmental variables, the main goals of the research are as follows (Figure 1): (1) mapping current and future (2040–2100) distributions across China under SSP126, SSP245, and SSP585 scenarios; (2) identifying the dominant environmental variables that govern habitat suitability; and (3) quantifying centroid shifts and proposing region-specific monitoring strategies.

## 2. Results and Interpretation

### 2.1. Model Accuracy Evaluation and Contribution of Environmental Variables

When modeling the potential distributions of four invasive *Amaranthaceae* species in China, optimized parameters were applied to enhance predictive accuracy. Specifically, *D. ambrosioides* was modeled using feature classes (FC) = PT (product and threshold) and a regularization multiplier (RM) = 1.5, *C. argentea* with FC = QPT (quadratic, product, and threshold) and RM = 0.9, *A. palmeri* with FC = LQT (linear, quadratic, and threshold) and RM = 1.2, and *A. spinosus* with FC = LQ (linear and quadratic) and RM = 0.3. The resulting model calibration achieved a Corrected Akaike Information Criterion Difference (ΔAICc) value = 0 for all species, indicating optimal parameter combination (Table 1).

**Figure 2:**
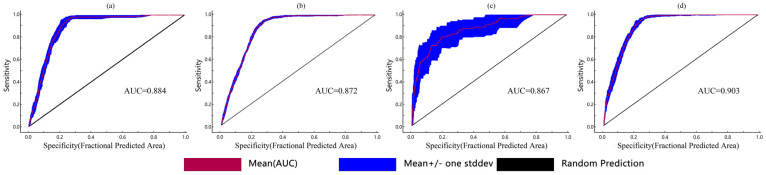
Under the current climatic conditions (1970–2000), the ROC curves and AUC values of four *Amaranthaceae* after running 10 times are as follows: (**a**) *D. ambrosioides*, (**b**) *C. argentea*, (**c**) *A. palmeri*, and (**d**) *A. spinosus*.

These optimized parameters were subsequently employed to simulate the habitat suitability of the four species across China. Post-optimization model validation demonstrated high discriminative capacity, with average AUC (area under the receiver operating characteristic curve) values of 0.884 for *D. ambrosioides*, 0.872 for *C. argentea*, 0.867 for *A. palmeri*, and 0.903 for *A. spinosus* for 10 repetitions (Figure 2 and Table 2). The closer the AUC value is to 1, the better the predictive effect of the MaxEnt model. Model validation showed high discriminative capacity, with mean test AUC values > 0.86 across all species.

**Figure 3:**
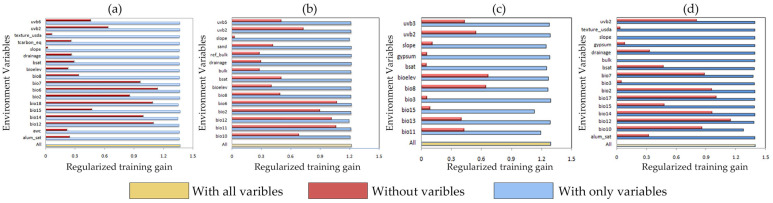
The importance of environmental variables evaluated by Jackknife testing: (**a**) *D. ambrosioides*, (**b**) *C. argentea*, (**c**) *A. palmeri*, and (**d**) *A. spinosus*.

The dominant environmental variables shaping habitat suitability for the four *Amaranthaceae* species were determined by the percentage contribution in the Jackknife test results (Figure 3) and are summarized in Table 3. Percentage contribution values, calculated as the mean of 10 independent model runs under current climatic conditions, identified the dominant predictors for each species. Variables with the highest contribution rates were classified as primary environmental variables affecting the potential distributions of the four invasive *Amaranthaceae* species.

### 2.2. Potential Distribution Under Current Climate Conditions

**Figure 4:**
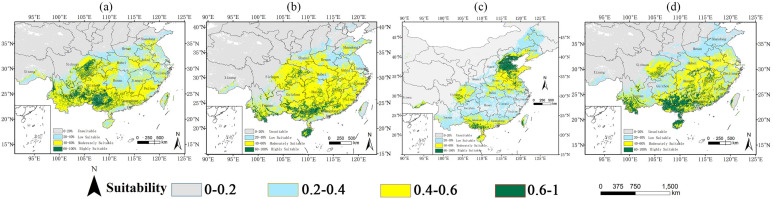
Current distribution of *Amaranthaceae* in China: (**a**) *D. ambrosioides*, (**b**) *C. argentea*, (**c**) *A. palmeri*, and (**d**) *A. spinosus*. Suitability: green = high (HSI > 0.6), yellow = moderate (0.4–0.6), and blue = low (0.2–0.4).

Model simulations revealed distinct biogeographic patterns among four invasive *Amaranthaceae* species, with *A. spinosus* predominantly distributed in northern China (e.g., Hebei Province), while the optimal habitats for *D. ambrosioides* (137.53 × 10^4^ km^2^), *C. argentea* (167.81 × 10^4^ km^2^), and *A. palmeri* (97.65 × 10^4^ km^2^) were concentrated in southern China. *D. ambrosioides* exhibited an expansion pattern radiating from the core high-suitability zones in Guangxi, Yunnan, and Sichuan, with moderate and low suitability areas encircling these areas. *C. argentea* demonstrated limited high-suitability coverage (23.13 × 10^4^ km^2^, 2.41% of China’s land area), primarily in Guangxi and Hainan. *A. palmeri* showed concentrated invasion risks in Yunnan, Guangdong, Guangxi, and Hainan (23.10 × 10^4^ km^2^ highly suitable habitat). Contrasting, the total suitable area of *A. spinosus* was 122.02 × 10^4^ km^2^, of which the high-suitability area was 30.83 × 10^4^ km2, with severe invasion trends in Hebei and Shandong provinces (Figure 4). The observed spatial configuration—characterized by continuous distribution ranges and disproportionately large, highly suitable areas—not only quantifies current invasion hotspots but also underscores the challenges in containment efforts, particularly given the family’s capacity for rapid range coalescence across heterogeneous landscapes.

### 2.3. Potential Distribution of Four Amaranthaceae Under Future Climate Conditions

#### 2.3.1. Potential Habitat for *D. ambrosioides* Under Climate Change Scenarios

**Figure 5:**
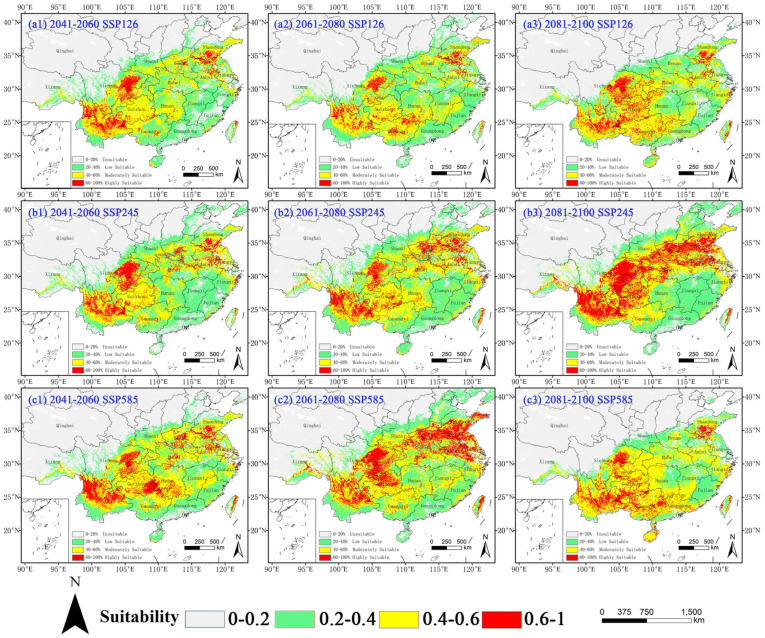
Potential suitable habitat of *D. ambrosioides* in China under future climate scenarios. Suitability: red = high (HSI > 0.6), yellow = moderate (0.4–0.6), and green = low (0.2–0.4).

It can be seen from Figure 5 and Table 4 that the habitat suitability of *D. ambrosioides* responds divergent across future climate scenarios. Under the SSP126 climate scenario, the total suitable area (≥0.4 probability) for *D. ambrosioides* declined by 6.79% (Δ = −9.34 × 10^4^ km^2^) by the 2050s, reaching minimum coverage (124.84 × 10^4^ km^2^, −9.23%) in the 2070s with 26.44% contraction in high-suitability zones before partial recovery to 142.14 × 10^4^ km^2^ (+3.35%) by the 2090s. Under the SSP245 climate scenario, the total suitable area (≥0.4 probability) for *D. ambrosioides* exhibited nonlinear dynamics, with an initial 7.09% reduction (2050s) followed by a 13.40% expansion (155.95 × 10^4^ km^2^) in the 2070s, culminating in a 151.80% surge in highly suitable habitat (66.21 × 10^4^ km^2^) and 23.68% total growth (170.10 × 10^4^ km^2^) by the 2090s. The SSP585 climate scenario demonstrated sustained invasion, with a 26.68% total expansion (174.22 × 10^4^ km^2^) and a 116.91% high-suitability growth (57.04 × 10^4^ km^2^) by the 2070s, forming continuous invasion corridors across the Yunnan–Guizhou–Sichuan and Huang–Huai regions. Spatial restructuring exhibited contrasting patterns between coastal habitat contraction and northward expansion, mechanistically driven by climate warming-induced latitudinal migration through thermal niche dynamics, with distribution shifts correlating with regional thermal regime modifications and precipitation seasonality gradients.

#### 2.3.2. Potential Habitat for *C. argentea* Under Climate Change Scenarios

**Figure 6:**
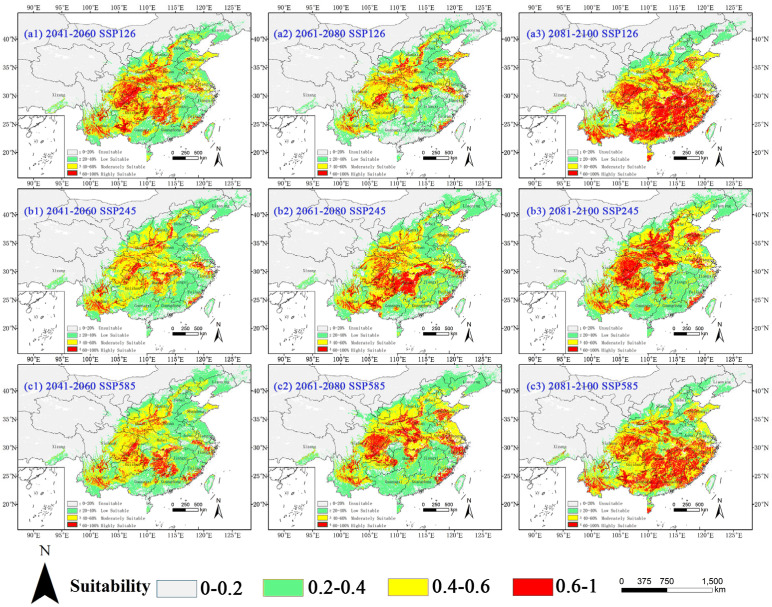
Potential suitable habitat of *C. argentea* in China under future climate scenarios. Suitability: red = high (HSI > 0.6), yellow = moderate (0.4–0.6), and green = low (0.2–0.4).

Our projections based on MaxEnt revealed substantial shifts in the suitable habitats of *C. argentea* under future climate scenarios (Figure 6 and Table 5). Under the SSP126 climate scenario, the total suitable area decreased by 5.54 × 10^4^ km^2^ by the 2050s compared to the current climate scenario, while high-suitability habitats showed remarkable expansion (a 105.6% increase). This trend intensified by the 2070s, with a 28.31% reduction in total suitable area (120.30 × 10^4^ km^2^), followed by a significant recovery (a 26.39% increase to 212.09 × 10^4^ km^2^) accompanied by exponential growth in high-suitability zones (a 284.40% increase) by the 2090s. The SSP245 climate scenario exhibited similar fluctuations, with total suitable area decreasing by 24.07 × 10^4^ km^2^ (2050s) and 8.71 × 10^4^ km^2^ (2070s), then rebounding to 173.79 × 10^4^ km^2^ (3.57% increase), with highly suitable habitat expanding 158.78% by the 2090s, particularly in northern China’s Henan, Hebei, Shandong, Shanxi, Shaanxi, and Beijing. Notably, the SSP585 climate scenario showed the most dramatic variations: successive reductions of 23.91 × 10^4^ km^2^ (2050s) and 15.37 × 10^4^ km^2^ (2070s) preceded a 33.3 × 10^4^ km^2^ surge by the 2090s, while high-suitability habitats demonstrated a 1.5–2.8-fold growth across all scenarios during terminal projections. This nonlinear response suggests temperature thresholds may trigger rapid expansion once critical thermal conditions are surpassed, though coastal habitat loss could reflect precipitation-induced mortality thresholds. The coastal contractions correlate with the exceedance of precipitation thresholds (Bio12 >2026 mm), inducing physiological drought stress.

The divergent temporal patterns between mid-century declines and late-century expansions highlight complex climate–vegetation interactions governing *C. argentea*’s distribution dynamics.

#### 2.3.3. Potential Habitat for *A. palmeri* Under Climate Change Scenarios

**Figure 7:**
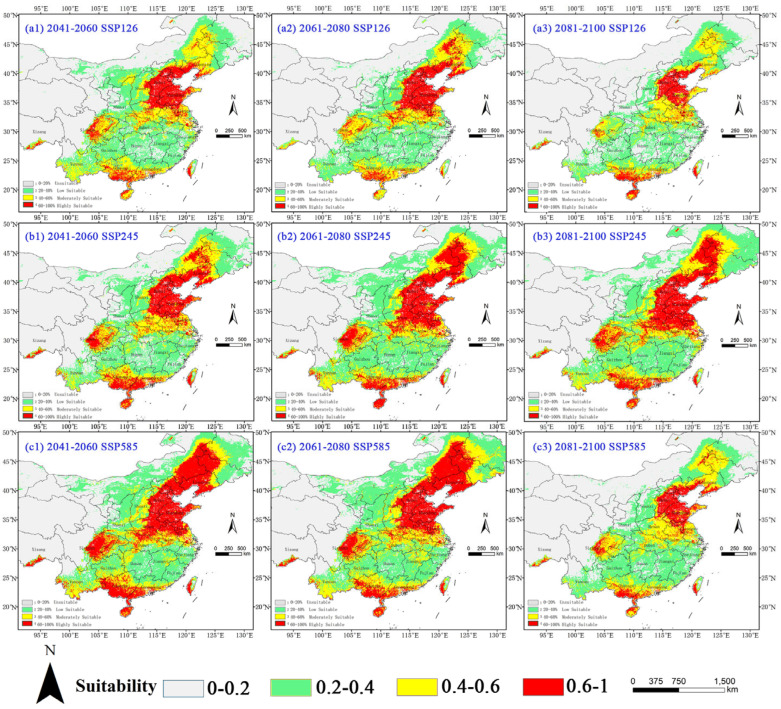
Potential suitable habitat of *A. palmeri* in China under future climate scenarios. Suitability: red = high (HSI > 0.6), yellow = moderate (0.4–0.6), and green = low (0.2–0.4).

Our MaxEnt projections demonstrate significant expansion of *A. palmeri*’s suitable habitats across all climate scenarios, with distinct spatial and temporal patterns (Figure 7 and Table 6).

Under the SSP126 climate scenario, the total suitable area of *A. palmeri* increased by 79.6 × 10^4^ km^2^ (81.50% growth) by the 2050s compared to current conditions, peaking at 156.25 × 10^4^ km^2^ (a 60.00% increase) in the 2070s before moderating to 125.67 × 10^4^ km^2^ (a 28.69% increase) by the 2090s, while high-suitability habitats maintained substantial growth rates (63.90–176.15%). The SSP245 climate scenario revealed exponential expansion, with the total habitat area increasing by 80.73% (176.49 × 10^4^ km^2^) by the 2050s and reaching 216.40 × 10^4^ km^2^ (a 121.60% increase) by the 2090s, accompanied by extraordinary high-suitability habitat growth rates of 247.51–367.92%, particularly in northeastern China (Heilongjiang, Jilin, Inner Mongolia, and Liaoning) and Yunnan. Most dramatically, the SSP585 projections showed 136.08% (230.55 × 10^4^ km^2^) and 147.61% (241.80 × 10^4^ km^2^) increases by the 2050s and 2070s, respectively, with high-suitability zones expanding 4.6-fold initially before stabilizing at 108.81% growth (a 54.21% total area increase) by the 2090s.

The accelerated invasion patterns in the North China Plain and Sichuan Basin correlate strongly with rising minimum winter temperatures, suggesting thermal thresholds may override altitudinal limitations to enable rapid colonization. Northward shifts (247 km under SSP245) reflect thermal niche release: warming reduces cold-induced mortality, enabling colonization beyond historical latitudinal limits. The three-fold to five-fold expansion of highly suitable habitat across scenarios underscores the species’ invasive potential under warming climates, though late-century stabilization under extreme SSP585 conditions implies possible physiological constraints at upper thermal limits.

#### 2.3.4. Potential Habitat for *A. spinosus* Under Climate Change Scenarios

**Figure 8:**
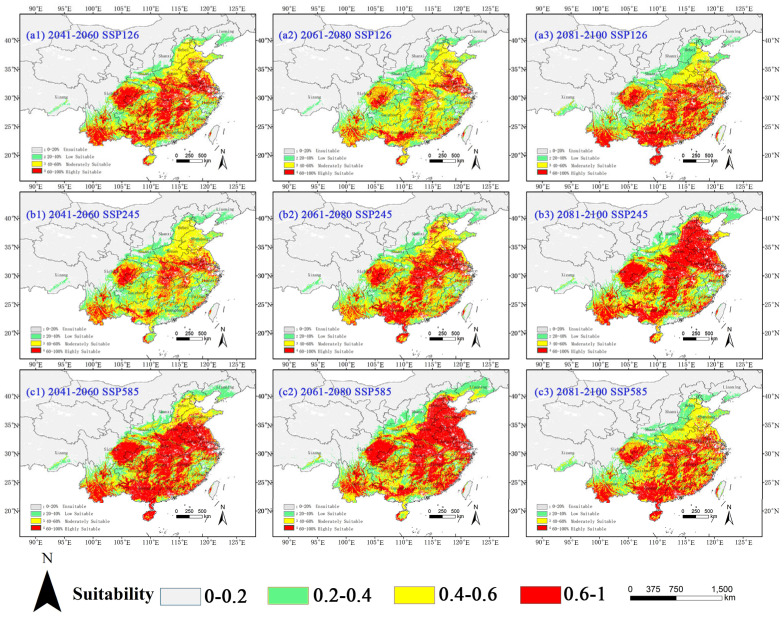
Potential suitable habitat of *A. spinosus* in China under future climate scenarios. Suitability: red = high (HSI > 0.6), yellow = moderate (0.4–0.6), and green = low (0.2–0.4).

The distribution patterns for *A. spinosus* under future climate scenarios are very different (Table 7 and Figure 8). Under the SSP126 climate scenario, the total suitable area increased by 61.55% (from 122.02 to 197.12 × 10^4^ km^2^) by the 2050s, peaking at 183.98 × 10^4^ km^2^ (a 50.78% increase) by the 2090s despite mid-century fluctuations. The SSP245 climate scenario exhibited accelerated growth in high-suitability zones, escalating from 68.5% (2050s) to an extraordinary 313.47% (2090s), with spatial expansion concentrated in the Henan, Jiangsu, and Shanxi provinces. Most dramatically, the SSP585 projections showed sustained expansion, with 76.30% (215.12 × 10^4^ km^2^) and 83.07% (223.38 × 10^4^ km^2^) total habitat increases by the 2050s and 2070s, respectively, though late-century growth moderated to 55.44% (189.67 × 10^4^ km^2^). This habitat optimization manifests through progressive conversion of low and moderate suitability areas to high-suitability zones, particularly clear in SSP585, where high-suitability habitats expanded 3.4–4.6-fold (339.44–360.46% increase) by the 2070s. Spatial analyses indicate northward shifts along the Yellow River Basin (Henan, Shandong), potentially driven by warming-enhanced thermal tolerance allowing the colonization of higher latitudes.

Collectively, these projections reveal divergent responses: all species exhibited significant net habitat expansion by the 2090s, though transient contractions in *D. ambrosioides* (SSP126-2070s: Δ = −9.23%) and *C. argentea* (SSP126-2070s: Δ = −28.31%) were also statistically significant. This nonlinearity underscores how thermal optima interact with precipitation thresholds to drive species-specific dynamics.

### 2.4. Centroid Shifts in Direction and Distance of Different Species

Under climate change, the distribution patterns of plant species have undergone significant shifts, with distribution centers exhibiting marked displacement [28].

**Figure 9:**
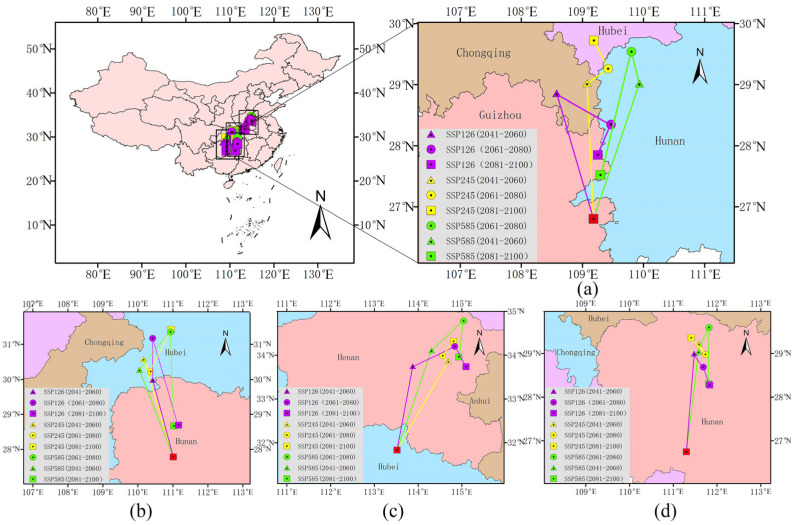
Total suitable habitat centroid distribution shifts for four *Amaranthaceae* under future climate scenarios: (**a**) *D. ambrosioides*, (**b**) *C. argentea*, (**c**) *A. palmeri*, and (**d**) *A. spinosus*.

The distributions of species have recently shifted to higher elevations at a median rate of 11.0 m per decade and to higher latitudes at a median rate of 16.9 km per decade. As is illustrated in Figure 9 and Table 8, the centroids of the four *Amaranthaceae* plants are predominantly concentrated in Guizhou, Henan, Hunan, and Hubei.

For *D. ambrosioides*, the centroid is currently centered in Tianzhu County, Guizhou (109°10′51.6″ E, 26°47′54.24″ N). Under the SSP126 climate scenario, its centroid migrates northwestward 236.44 km to Youyang County, Chongqing, by the 2050s, then shifts southeast 103.89 km to Huayuan County, Hunan (109°15′07.2″ E, 27°50′59.28″ N) in the 2070s, culminating in a 59.30 km southward retreat to Tongren City, Guizhou by the 2090s. In the SSP245 scenario, a northward migration of 247.76 km to Youyang County is followed by incremental northward shifts into Hubei Province. The most extreme response occurs under SSP585, featuring a 258.03 km northeast leap to Yongshun County, Hunan, before a 229.69 km southward rebound to the Wanshan District, Guizhou (109°17′45.6″ E, 27°31′15.96″ N).

For *C. argentea*, the current distribution center of *C. argentea* originates in Xinhua County, Hunan (111°0′43.2″ E, 27°46′44.76″ N). Under the SSP126 scenario, the center migrates northwest by 251.64 km to Wufeng County, Hubei (110°25′26.4″ E, 31°10′35.4″ N) by the 2050s and continues northward by 132.53 km to Badong County in the 2070s, which is followed by a 284.98 km southward reversal in the 2090s. Under the SSP585 scenario, the center moves northwest by a 292.44 km shift to Jianshi County, Hubei (110°2′34.8″ E, 30°16′14.16″ N) in the 2050s, with subsequent 147.75 km northeast and 298.79 km southward movements, ultimately reaching Yuanling County, Hunan.

For *A. palmeri*, the current distribution center originates in the Zengdu District, Hubei (113°31′4.800″ E, 31°49′47.280″ N), with future trajectories confined to Henan Province. Under the SSP245 scenario, there is a northward displacement of 250.98 km to Xihua County (114°41′20.400″ E, 33°51′46.440″ N), followed by 17.91 km westward and 42.98 km eastward adjustments in the 2070s. Under the SSP585 scenario, the center migrates northeast by 264.12 km to Fugou County, preceding a 91.73 km southward retreat to Taikang County (114°55′44.400″ E, 33°57′49.320″ N).

For *A. spinosus*, the current distribution center is in Shaoyang County, Hunan Province (111°17′56.400″ E, 26°44′48.480″ N). In the SSP245 scenario, the longest single-stage northward migration of 276.73 km to the Dingcheng District (111°35′27.600″ E, 29°13′25.320″ N) occurs in the 2050s, while a 256.32 km northeast advance to Taoyuan County is followed by a 143.29 km southward withdrawal to Anhua County in the SSP585 scenario. According to the statistical result, over 68% of endpoints are clustered within the Wuling Mountain transitional belt (27° N–29° N), underscoring elevation-dependent constraints.

### 2.5. Niche Divergence Supports Species-Specific Plasticity

**Figure 10:**
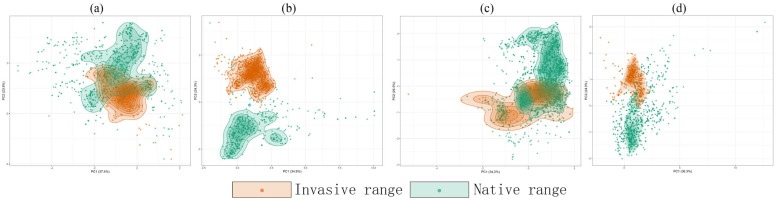
Niche overlap plot for four *Amaranthaceae* between native range and invasive range: (**a**) *D. ambrosioides*, (**b**) *C. argentea*, (**c**) *A. palmeri*, and (**d**) *A. spinosus*.

While *D. ambrosioides* maintains high stability (97.18%), its substantial unfilling rate (54.07%) and moderate niche overlap (Schoener’s D = 0.269) suggest incomplete utilization of its native niche. *C. argentea* achieves invasion through extreme niche reorganization (Schoener’s D = 0.0064), though its high unfilling (91.54%) reveals significant invasion costs. *A. spinosus* exhibits explosive niche expansion (expansion rate: 97.3%, *p* = 0.07), with synchronously high unfilling (97.3%), indicating intense environmental filtering during invasion. In contrast, *A. palmeri* demonstrates exceptional niche stability (99.35%) and minimal expansion (0.65%), supported by significant niche equivalency (*p* = 0.0297), establishing it as a paradigm of climate matching (Table 9). PCA ordination (Figure 10) further visualizes these dynamics: *A. spinosus*’s 97.3% expansion corresponds to large-scale displacement in temperature-precipitation space, while *C. argentea*’s low Hellinger’s I value (0.0532) confirms fragmented niche distribution.

## 3. Discussion

### 3.1. Model Accuracy Analysis

The reliability of species distribution models depends critically on appropriate environmental variable selection and avoidance of overfitting from redundant occurrence data [30]. To address these challenges, we first applied Pearson correlation analysis (|r| > 0.8) to remove collinear variables, followed by ENMTools filtering to eliminate predictors with low contribution importance (<1%) and spatially clustered occurrence points. Given the risks of over-fitting associated with default parameter, we optimized model complexity using the Ecological Niche Model Evaluation (ENMeval) package (V 4.3.2) through species-specific tuning of regularization multipliers (1–4) and feature classes [31]. This yielded ΔAICc = 0 for all four *Amaranthaceae* plants, effectively achieving model accuracy. Reconstructed models using optimized parameters exhibited robust predictive performance, with mean AUC values exceeding 0.86 across all species (0.867–0.930). While our framework incorporated multi-dimensional predictors (climate, soil, elevation, and solar radiation), inherent limitations of this metric persist [32]. Model projections may overestimate climate-driven suitability in regions with active human-mediated dispersal. Moreover, 4.63% of *Amaranthaceae* occurrence records lacked soil metadata, and equal-interval classification may obscure species-specific ecological thresholds despite enabling cross-species comparison. Future model iterations should integrate anthropogenic covariates like road density and crop trade volumes. Additionally, equal-interval classification may not capture species-specific ecological thresholds, though it allows consistent cross-species comparison. Nevertheless, the ΔAICc = 0 optimization ensures robustness in identifying climate-driven distribution trends, and parameter tuning mitigates residual concerns—though mechanistic field validation remains essential.

### 3.2. The Key Environmental Drivers Influencing Amaranthaceae Invasion

The key environmental variables affecting four invasive *Amaranthaceae* species were filtered by Jackknife and Pearson’s correlation [33]. In this study, Bio6 and Bio11 emerged as dominant drivers, explaining >50% of the distribution variance. This aligns with known physiological constraints: *Amaranthaceae* species exhibit winter mortality below species-specific thresholds. In detail, Bio6 exhibited high permutation importance for *D. ambrosioides* (44.6%) and *C. argentea* (18.4%), while Bio11 contributed 56.4% and 15.7% to *C. argentea* and *A. palmeri*, respectively. On average, high-latitude or high-altitude habitats are consistently colder than low-latitude or low-altitude ones at any time during the year [34]. Positive correlations between Bio6/Bio11 and occurrence probabilities demonstrated that thermal requirements critically govern their latitudinal expansion. Among precipitation variables, Bio12 showed maximal influence on *D. ambrosioides* (33.3%), with peak suitability occurring at 1076.12–1956.18 mm. Elevation dominated *A. palmeri*’s distribution (27.3%), exhibiting optimal suitability below 256.36 m. Soil properties played secondary roles, with BSAT and DRAINAGE explaining 12.9% and 13.7% of *A. spinosus*’ habitat suitability, respectively, within thresholds of 6.5–63.82% and 0.6–1.02. UVB-2 exerted moderate effects on *A. palmeri* (16.2%) and *A. spinosus* (10%), with shared suitability at 0.67–1.36 kJ/m^2^. Multivariate interaction analyses revealed synergistic temperature–elevation–precipitation effects driving habitat shifts.

### 3.3. Suitable Habitat and Its Dynamics Change

Based on the optimized MaxEnt model, we predicted the potential suitable habitats for four *Amaranthaceae* species, providing foundational research for assessing future climate change impacts on invasive plant distributions [35]. Our projection of a significant northward shift in suitable habitats (mean velocity: 16.3 km/decade) aligns with reported shifts for 74% of Chinese invasive plants and supports niche conservatism theory, as these C4 species retain ancestral thermal tolerances while exploiting warming-induced range expansions [36]. The expansion rates are likely attributable to enhanced C4 photosynthetic efficiency under elevated CO_2_ [23], potentially accelerating invasion in the North China Plain—a region analogous to their native arid habitats. Competitive release may further facilitate expansion, as native C3 plants exhibit reduced productivity under high-temperature extremes [34]. Under the SSP126 scenario, the high-suitability area for *D. ambrosioides* declined during the 2050s and 2070s but rebounded significantly by the 2090s, retaining 82.6% of its baseline extent with a 20.8% increase in newly added area. Conversely, *C. argentea* experienced only a short contraction in the 2070s, recovering to retain 99.6% of its baseline area by the 2090s and expanding by 46.7 × 10^4^ km^2^. The overall suitable habitat areas for *A.palmeri* and *A.spinosus* increased by 81.5% and 61.5% in the 2050s, respectively, indicating a continuous expansion trend. This suggests that under low emissions, suitable habitats for these *Amaranthaceae* species progressively expanded through niche redistribution. Notably, *A. palmeri*’s projected 247% (SSP245, 2050s) to 460% (SSP585, 2050s) growth in high-suitability habitat far exceeds the 21% range expansion modeled for *Ambrosia artemisiifolia* by 2050 [29], highlighting its exceptional invasion vigor.

**Figure 11:**
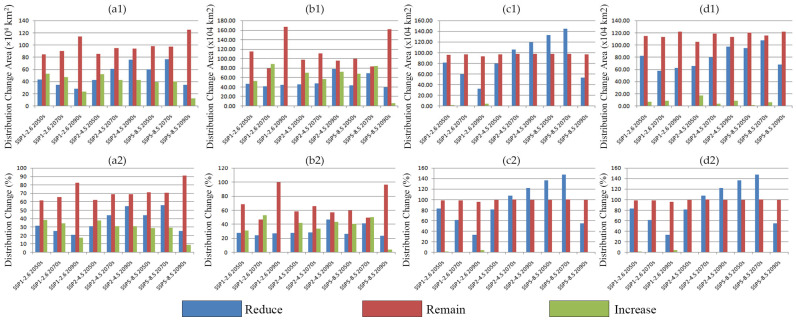
Distribution change of four *Amaranthaceae* species in China: (**a**) *D. ambrosioides*, (**b**) *C. argentea*, (**c**) *A. palmeri*, and (**d**) *A. spinosus*.

Habitat dynamics diverged significantly under higher emission scenarios (SSP245 and SSP585). Under SSP245, the retention rate of existing high-suitability habitats declined for all species, but the conversion rate of low- to medium-suitability areas into high-suitability zones increased. Under SSP585, the newly added habitat exceeded that under lower emissions. While the retention rates for *D. ambrosioides* and *C. argentea* exhibited considerable fluctuations, their distributions still expanded overall. In contrast, *A. palmeri* and *A. spinosus* underwent explosive growth in the 2070s, increasing by 460.8% and 360.5%, respectively, with new suitable areas concentrated primarily in the Huang–Huai Plain and northern North China Plain (Figure 11). This study reveals that temperature-driven niche expansion dominated the poleward shift under high emissions. The contraction of *D. ambrosioides* under SSP126 reflects precipitation limitations surpassing warming benefits, while the high-suitability growth amid total area declines of *C. argentea* suggests anthropogenic dispersal compensates for marginal climate suitability. Nevertheless, long-term instability in habitat retention rates highlights the complex regulatory mechanisms of climate change on the distributions of these invasive species [37]. This pronounced and taxon-specific expansion, particularly under high emissions, underscores the critical need for incorporating taxon-specific risk assessments within national biosecurity frameworks.

In the research, the plant migration for all four *Amaranthaceae* species is influenced by changes in temperature and precipitation [35], with their distribution centers shifting towards higher latitudes. This substantial latitudinal displacement aligns with global patterns of species migration as climate adaptation [38] and will escalate agricultural conflicts in China’s ‘Corn Belt’. The 149-fold disparity in expansion rates between *A. spinosus* (97.3%) and *A. palmeri* (0.65%) establishes a plasticity gradient governing synergistic invasion. Highly plastic species (*A. spinosus* and *C. argentea*) create invasion beachheads through niche reorganization (Hellinger’s I = 0.0532) and environmental tolerance broadening (unfilling >90%), enabling conservatives (*A. palmeri* and *D. ambrosioides*) to establish via climate-matched corridors. Divergent migration rates (e.g., *A. palmeri* shifting 247 km northward under SSP245 vs. *D. ambrosioides*’ contraction) reveal trait-mediated climate vulnerabilities—a critical gap in single-species models. Centroid shifts to critical latitudes intensify crop competition: *A. palmeri* reduces soybean yields by 79%, while *D. ambrosioides*’ allelopathic compounds suppress wheat germination. The North China Plain (34–40° N) emerges as a high-risk invasion corridor, where agricultural imports amplify invasion hotspots projected to incur additional losses of CNY 1.2 billion/year by 2050. Furthermore, their incursion into the Yellow River Basin threatens riparian ecosystems, where native *Astragalus sinicus* already faces habitat fragmentation. Consequently, these projections necessitate revising China’s Invasive Species Management Plan (2023–2030) to prioritize surveillance in the 34–40° N latitudinal band, with preemptive herbicide deployment in Henan and Shaanxi, integrated with the National Phytosanitary Database for real-time alerts.

### 3.4. Endangered Status and Prevention Recommendations

Our prediction from this study indicates a critical escalation in the invasion potential of four *Amaranthaceae* species. The dramatic increase in imported grains, international mail, and inbound travelers has facilitated the establishment of these species (e.g., *A. palmeri*) as dominant populations within agricultural ecosystems. Concurrently, ongoing urbanization has accelerated their invasion into built-up urban areas [39], likely driven by significant niche overlap between invaded and native ranges [40]. The explosive expansion of *A. palmeri* in the North China Plain (SSP585) correlates with both winter warming and grain import corridors, while the recovery in the 2090s (SSP245) of *D. ambrosioide* coincides with medicinal cultivation zones, suggesting synergistic climate-anthropogenic drivers. In conclusion, the Yunnan–Guizhou–Sichuan region, the Yellow River Basin, and the North China Plain were identified as severely affected zones.

Our identification of ‘invasion corridors’ in the Huang–Huai Plain (112–120° E, 33–36° N) enables targeted Early Detection Rapid Response (EDRR). Integrated drone surveillance and farmer citizen science have reduced monitoring costs by 30%. In established invasion areas, we recommend implementing manual removal coupled with native vegetation restoration programs [41], supplemented by species-optimized interventions: *D. ambrosioides* control via 4:1 intercropping with *Tagetes erecta*, achieving 68% biomass reduction through terthienyl allelopathy, coupled with medicinal valorization for hepatocellular carcinoma prevention under *Phytolacca americana*-level containment protocols [42]; *C. argentea* suppression through pheromone traps targeting seed–weevil vectors identified ornamental trade hubs; and *A. palmeri* management through pre-emergence rotation of acetochlor (1.2 kg/ha) and glufosinate (0.8 kg/ha), supplemented by 20 cm-deep tillage prior to seed maturation. However, overuse risks selecting dual-resistant biotypes, necessitating mandatory monthly qPCR monitoring of EPSPS mutations during the growing season and *A. spinosus* replacement via *Astragalus sinicus* cropping, with biomass conversion to 14% crude protein silage, generating USD 120/ha revenue while reducing weeding costs by 40%—an approach strategically aligned with China’s ‘green agriculture’ subsidies.

For emerging risk zones—particularly ports, grain import terminals, processing facilities, and transportation corridors (rail/road)—we propose establishing climate-informed surveillance systems in Hunan, Hubei, and Henan concurrently, integrating the real-time phytosanitary databases tracking *Amaranthaceae* dispersal [43] and the early-detection rapid-response (EDRR) frameworks targeting northward-expanding populations [44]. Controlling invasions at these anthropogenic-natural interfaces will disrupt dispersal pathways while addressing range shifts identified under climate scenarios.

## 4. Conclusions

This study demonstrates that temperature variables (Bio6 and Bio11) are the primary drivers of habitat expansion for four invasive *Amaranthaceae* species in China, collectively explaining >50% of the distribution dynamics. Under climate change, all species exhibited significant northward shifts in their distribution centroids, with *A. palmeri* migrating up to 247 km poleward under SSP245 scenarios. While net habitat expansion occurred by the 2090s, transient contractions in *D. ambrosioides* and *C. argentea* under mid-century scenarios reveal vulnerability to precipitation constraints and dispersal limitations. Crucially, high-risk invasion corridors were identified in the North China Plain (34–40° N) and the Yellow River Basin, where *A. palmeri*’s highly suitable habitat expanded by 367%. These findings underscore the urgent need for latitude-specific monitoring strategies targeting thermally vulnerable zones, directly informing China’s National Invasive Species Management Plan (2023–2030).

## 5. Materials and Methods

### 5.1. Species Data Source

**Figure 12:**
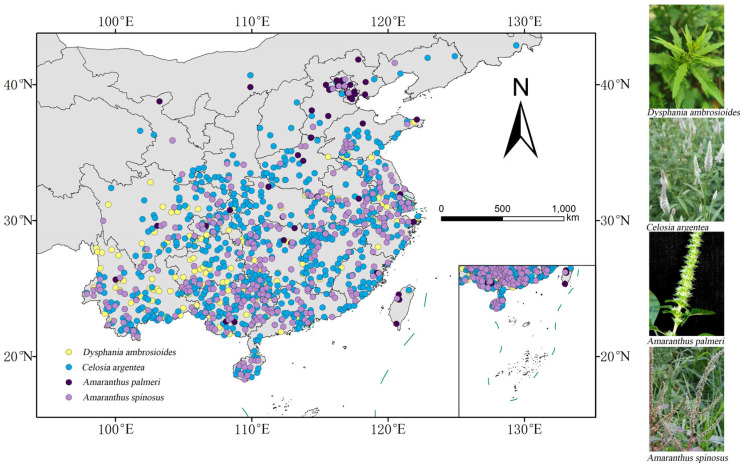
Distribution sites of four *Amaranthaceae* species in China (*D. ambrosioides*, *C. argentea*, *A. palmeri*, and *A. spinosus*).

The accuracy of MaxEnt model simulations and predictions of potential species distribution areas is critically dependent on the quality of species occurrence data. Wild occurrence records for the four *Amaranthaceae* species (*D. ambrosioides*, *C. argentea*, *A. palmeri*, and *A. spinosus*) were collected by the following steps: (1) Internet retrieval: online database retrieval from the Global Biodiversity Information Facility (GBIF) [45], and the Chinese Virtual Herbarium (CVH, http://www.cvh.ac.cn/, accessed 10 April 2025) [46]. (2) Literature retrieval: supplemented by a literature review of the China National Knowledge Infrastructure (CNKI, http://www.cnki.net/, accessed 15 April 2025) using the respective species names as search terms; all locality descriptions were meticulously verified for village-level precision, and missing coordinates for records with detailed descriptions were derived using an online geo-referencing tool (https://jingweidu.bmcx.com/, accessed 17 April 2025). (3) Data duplication: to mitigate spatial autocorrelation and potential model over-fitting, distribution points and environmental variables were imported into ArcGIS (v10.8). We applied a 7.5 km buffer radius around each occurrence point using the Buffer tools in ArcGIS, as 7.5 km optimally balanced spatial independence and sample retention. Points within overlapping buffers were reduced to one record per cluster via systematic random selection, and further refinement was performed using the ‘trim duplicate’ function in ENM Tools to eliminate spatially coincident points. (4) Sample size: ultimately, we gathered 202 occurrence records for *D. ambrosioides*, 677 occurrence records for *C. argentea*, 48 occurrence records for *A. palmeri*, and 306 occurrence records for *A. spinosus* (Figure 12), and the final file was converted into CSV format for subsequent analysis.

### 5.2. Environmental Variables and Processing

The number of environmental variables significantly impacts the complexity of the MaxEnt model [47]. Climate is widely recognized as a primary determinant of species distributions [32], with climate change potentially causing habitat displacement or even loss. Topography, soil properties, and solar radiation also significantly influence species’ geographic ranges. For this simulation, 53 environmental variables (climate, topography, soil, and solar radiation) were utilized. Current-period (1970–2000) and future-period (2050s: 2041–2060; 2070s: 2061–2080; 2090s: 2081–2100) climate data and elevation data were obtained from WorldClim v2.1 [48] (http://www.worldclim.org; accessed 20 February 2025). Future projections employed SSP using the CMIP6 model under three scenarios [49]: SSP126 (low emissions), SSP245 (medium emissions), and SSP585 (high emissions) [50]. Aspect data were derived from elevation using ArcGIS spatial tools. Soil variables (26 parameters) were sourced from the Harmonized World Soil Database (HWSD v1.2; http://www.fao.org/soils-portal; accessed 20 April 2025). Global UV-B radiation data (UVB1–UVB6) were acquired from the Global UV-B Radiation Database [51] (http://www.ufz.de/gluv/; accessed 21 April 2025).

To minimize over-fitting induced by multicollinearity [52], environmental variables were filtered through sequential screening: First, species occurrence data and 53 variables were processed in MaxEnt (default settings) to calculate percentage contribution and permutation importance [53]. Variables with a contribution < 1% and permutation importance < 1% were excluded, while values at occurrence points were extracted using ArcGIS 10.8’s Resample tool. Second, Pearson correlation analysis was conducted in SPSS 28.0 (IBM Corp., USA), with correlation matrices visualized via heatmap in R. When |r| was >0.8 between paired variables, the variable with the lower MaxEnt contribution was systematically removed [54].

Dominant variables were identified via MaxEnt’s permutation importance [55] (Table 10). For *D. ambrosioides*, the min temperature of the coldest month (Bio6, with a 44.6% contribution rate) was identified as the most influential environmental variable. Meanwhile, Annual Precipitation (Bio12, 33.3% contribution rate) and Precipitation of Warmest Quarter (Bio18, 9.5% contribution rate) were also influential predictors. By observing the response curve, the occurrence probability of *D. ambrosioides* exhibited a near-linear positive correlation with these variables, showing no marked decrease at higher values. Response curve analysis revealed critical thresholds for habitat suitability: occurrence probabilities fell below 0.01 when Bio6 < −17.52 °C, annual precipitation (Bio12) < 679.23 mm, or precipitation of the warmest quarter (Bio18) < 330.72 mm. Therefore, the optimal habitat conditions were defined as follows: Bio6 ranged from 3.50 to 10.25 °C, Bio12 ranged from 1076.12 to 1956.18 mm, and Bio18 ranged from 593.96 to 991.04 mm, representing climatically stable zones conducive to population establishment.

By analyzing the impact of environmental variables on the distribution of *C. argentea* (Table 10), the mean temperature of the coldest quarter (Bio11, with a 56.4% contribution) was identified as the most influential environmental variable governing the distribution of *C. argentea*. Additional contributing factors included the minimum temperature of the coldest month (Bio6, with an 18.4% contribution), mean temperature of the warmest quarter (Bio10, with a 6.5% contribution), and annual precipitation (Bio12, with a 7.1% contribution), with cumulative ecological relevance. Response curve analyses revealed a continuous positive correlation between presence probability and increasing values of both Bio6 and Bio11. Conversely, presence probability exhibited threshold-dependent declines when Bio10 and Bio12 exceeded critical values. The species’ occurrence probability rose continuously with higher Bio6 (>0.14 °C) and Bio11 (>4.96 °C) values. Bio10 >16.63 °C initially favored occurrence, but suitability collapsed near 28.94 °C before recovering at higher temperatures. The annual precipitation exceeding 2026.22 mm (Bio12) reduced the probability to 0.23, indicating critical hydrological constraints. Optimal habitat parameters were delineated as follows: Bio6 ranged from 0.14 to 23.10 °C, Bio10 ranged from 16.63 to 35.48 °C, Bio11 ranged from 4.96 to 26.79 °C, and Bio12 ranged from 1097.36 to 2026.22 mm.

For *A. palmeri*, elevation (27.3% contribution) was identified as the primary environmental determinant governing the distribution, with significant contribution from UV-B seasonality (UVB2, with a 16.2% contribution), mean temperature of the coldest quarter (Bio11, with a 15.7% contribution), precipitation seasonality (Bio15, with a 12.7% contribution), and mean temperature of the wettest quarter (Bio8, with a 12.3% contribution). Response curves indicated progressive declines in occurrence probability with increasing elevation (when elevation exceeded 256.36 m, probability reduced below 0.5; when elevation exceeded 918.29 m, probability collapsed to 0.2) and UVB2 values, while showing positive correlations with Bio8, Bio11, and Bio15. Optimal habitat conditions were confined to elevations below 256.36 m combined with UVB2 levels of 0.65–1.36, demonstrating strict physiological constraints mediated by altitudinal gradients and photoperiodic sensitivity. Bio8 ranged from 24.42 to 37.49 °C, Bio11 ranged from −1.11 to 24.91 °C, and Bio15 ranged from 112.79 to 160.44%.

For *A. spinosus*, habitat suitability was primarily governed by mean temperature of warmest quarter (Bio10, with a 25.2% contribution) and temperature annual range (Bio7, with a 20.8% contribution), with secondary influences from soil drainage (a 13.7% contribution), base saturation (BSAT, with a 12.9% contribution), and UV-B seasonality (UVB2, with a 10.0% contribution). Response curve analysis demonstrated a positive linear relationship between occurrence probability and Bio10, contrasting with negative correlations for Bio7, drainage, BSAT, and UVB2. Physiological thresholds were identified at Bio7 > 36.11 °C and Bio10 < 22.34 °C, where occurrence probability declined to 0.2, representing critical thermal exclusion boundaries. Optimal growth conditions occurred within Bio7 (8.90–27.78 °C), Bio10 (26.06–36.10 °C), BSAT (6.5–63.82%), drainage (0.6–1.02), and UVB2 (0.67–12.66), reflecting this species’ adaptation to sustained warm-season temperatures while exhibiting sensitivity to excessive annual thermal variability, suboptimal soil chemistry, and photoperiodic extremes.

### 5.3. Formatting of Mathematical Components

In this study, MaxEnt parameters were systematically optimized to mitigate over-fitting by using the ENMeval data package [56]. FC and RM were jointly calibrated. Through exhaustive evaluation of 31 FCs within R v3.6.3, optimal parameters were selected from 1160 candidate models. Model performance was assessed via corrected ΔAICc, with the configuration achieving ΔAICc = 0 identified as optimal [57].

Model robustness was ensured through randomized partitioning: 75% of the occurrence records trained the models, while 25% validated predictions [53]. Under optimized parameters, 10 replicate runs were executed under different climate scenarios. Final potential distribution maps were generated by averaging ASCII-format outputs. The accuracy was quantified by using the AUC, with performance classified as good in 0.8 ≤ AUC < 0.9 and excellent in AUC ≥ 0.9 [58], and the TSS, whose threshold was determined by R software, with values > 0.6 indicating good performance. While high AUC values (>0.8) indicate robust relative performance, they should be interpreted alongside the ecological realism of projections.

Following reclassification with the Habitat Suitability Index (HSI), outcomes were sorted by ArcGIS 10.8 into four suitability types: unsuitable (0–0.2), low suitability (0.2–0.4), moderate suitability (0.4–0.6), and high suitability (0.6–1.0) [59], which aligns with published invasion studies in which field validation confirmed a > 60% occurrence frequency at HSI > 0.6 [53]. Spatial extents for each class were quantified through sequential processing: pixel counts per suitability tier were extracted from raster attribute tables, total areal coverage was calculated by multiplying pixel counts by grid resolution, and values were scaled to China’s terrestrial landmass (9.6 × 10^6^ km^2^) using Microsoft Excel.

### 5.4. Centroid Change Analysis

To quantify spatiotemporal changes in suitable habitats for the four *Amaranthaceae* species, we defined grid cells with occurrence probability ≥ 0.5 as potentially suitable areas [60]. Using the “Distribution changes between binary SDMs” tool in the SDM toolbox [38], future projections under climate scenarios were spatially overlaid and compared against current conditions. Based on the binary suitability matrix method, habitat transitions were classified into three categories: expansion areas (0 → 1), stable areas (1 → 1), and contraction areas (1 → 0) [61]. Spatial change patterns were visualized in ArcGIS 10.8. Additionally, centroid coordinates of suitable habitats representing core distributional centers were calculated using the SDM toolbox in ArcGIS to quantify directional shifts [62]. Migration distances were derived from centroid displacements between current and future periods, characterizing spatial trajectories of range adjustments.

### 5.5. Quantifying Niche Shifts Between Native and Invaded Ranges

We employed ENMtools to assess niche dynamics for four key Amaranthaceae invaders (*D. ambrosioides*, *C. argentea*, *A. palmeri*, and *A. spinosus*). Environmental layers were clipped to native backgrounds and invaded backgrounds. Niche equivalency (100 permutations) and similarity tests (background randomization) were conducted to calculate metrics including Schoener’s D, stability, and expansion.

## Figures and Tables

**Table 1:** The optimized parameters and evaluation metrics for the MaxEnt model.

Species	*D. ambrosioides*	*C. argentea*	*A. palmeri*	*A. spinosus*
Optimized FC	PT	QPT	LQT	LQT
Optimized RM	1.5	0.9	1.2	0.3
ΔAICc	0	0	0	0
Omission rate (5%)	0.22	0.054217	0.166666	0.039473
W-AICc	1.34 × 10^−3^	1.10 × 10^−3^	1.72 × 10^−3^	9.11 × 10^−4^
TSS	0.6804	0.7057	0.712	0.7274
Calibration figure	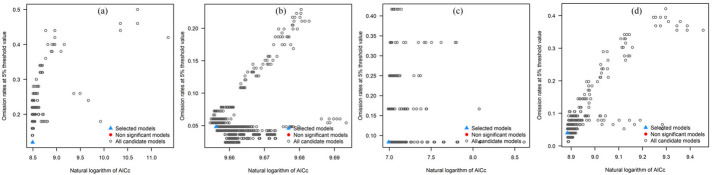			

Note: FC represents feature class; RM represents regularization multiplier.

**Table 2:** The AUC values of the four *Amaranthaceae* species after 10 replicates under current climatic conditions.

Species	AUC Type	1	2	3	4	5	6	7	8	9	10	Avg.
*D. ambrosioides*	Training AUC	0.9287	0.9348	0.9303	0.9286	0.934	0.9277	0.9279	0.9286	0.9324	0.929	0.9302
	Test AUC	0.897	0.8588	0.8854	0.8708	0.8714	0.8899	0.8978	0.8923	0.8836	0.8929	0.884
*C. argentea*	Training AUC	0.8986	0.902	0.9016	0.9036	0.9009	0.9017	0.9	0.9032	0.9015	0.9005	0.9014
	Test AUC	0.8793	0.8705	0.8693	0.8648	0.8774	0.8734	0.8741	0.8683	0.8721	0.8715	0.8721
*A. palmeri*	Training AUC	0.9153	0.9354	0.9197	0.9032	0.921	0.9239	0.9081	0.9227	0.9139	0.8975	0.9161
	Test AUC	0.8532	0.8131	0.8874	0.923	0.9008	0.7818	0.7663	0.874	0.9309	0.9443	0.8675
*A. spinosus*	Training AUC	0.9118	0.9151	0.9167	0.911	0.9076	0.9093	0.9118	0.9111	0.9138	0.9145	0.9123
	Test AUC	0.9073	0.8932	0.8898	0.9052	0.9206	0.9155	0.8994	0.9079	0.9033	0.8942	0.9036

**Table 3:** Contribution rates of environmental variables (%).

Variable		Description	Contribution (%)			
			*D. ambrosio* *ides*	*C. argentea*	*A. palmeri*	*A. spino* *sus*
Climate	Bio2	Mean Diurnal Range	—	0.9	—	9.5
	Bio3	Isothermality (Bio2/Bio7)(× 100)	—	—	0.2	2.4
	Bio6	Min. Temperature of Coldest Month	44.6	18.4	—	—
	Bio7	Temperature Annual Range	2.9	—	—	20.8
	Bio8	Mean Temperature of Wettest Quarter	0.6	0.8	12.3	—
	Bio10	Mean Temperature of Warmest Quarter	—	6.5	—	25.2
	Bio11	Mean Temperature of Coldest Quarter	—	56.4	15.7	—
	Bio12	Annual Precipitation	33.3	7.1	—	1.7
	Bio13	Precipitation in Wettest Month	—	—	1.9	—
	Bio14	Precipitation in Driest Month	0.6	—	—	0.7
	Bio15	Precipitation Seasonality	0.5	—	12.7	0.5
	Bio18	Precipitation of Warmest Quarter	9.5	—	—	—
Altitude	elev	Elevation	0.8	1.9	27.3	—
	slope	Slope Degree	1.3	2.5	5.2	—
Soil	ALUM_SAT		0.6	—	—	1.1
	AWC		0.5	—	—	—
	BSAT		0.7	0.6	3.3	12.9
	DRAINAGE		0.8	0.9	—	13.7
	GYPSUM		—	—	1.7	—
	TCARBON_EQ		0.5	—	—	—
	TETURE_USDA		0.6	—	—	1
	BULK		—	0.6	—	—
	REF_BULK	Reference Bulk Density	—	0.5	—	—
UV-B	UVB2	UV-B Seasonality	0.7	1.6	16.2	10
	UVB3	Mean UV-B of Highest Month	—	—	3.6	—
	UVB5	Sum of Monthly Mean UV-B during Highest Quarter	—	1	—	—
	UVB6	Sum of Monthly Mean UV-B during Lowest Quarter	0.8	—	—	—

Note: ‘— ’represents variables not selected for MaxEnt models.

**Table 4:** Suitable area of *D. ambrosioides* in different periods (×10^4^ km^2^).

Period		Low Suit	Change%	Middle Suit	Change%	High Suit	Change (%)	Total Suit	Change%
Current		83.67	—	111.23	—	26.30	—	137.53	—
SSP126	2050s	114.15	36.42	104.03	−6.48	24.16	−8.12	128.19	−6.79
	2070s	121.23	44.89	105.49	−5.16	19.34	−26.44	124.84	−9.23
	2090s	89.38	6.82	113.89	2.38	28.25	7.43	142.14	3.35
SSP245	2050s	120.92	44.52	99.61	−10.45	28.17	7.14	127.78	−7.09
	2070s	115.56	38.11	121.92	9.60	34.04	29.44	155.95	13.40
	2090s	111.18	32.87	103.89	−6.61	66.21	151.80 ↑	170.10	23.68
SSP585	2050s	98.13	17.28	122.59	10.21	35.49	34.95	158.08	14.94
	2070s	112.84	34.86	117.18	5.35	57.04	116.91	174.22	26.68
	2090s	76.31	−8.80 ↓	131.34	18.07	28.32	7.70	159.66	16.09

Note: “Change (%)” represents the percentage change in suitability area compared to the current period, “↑” indicates an increase (maximal growth), “↓” indicates a decrease (maximal reduction).

**Table 5:** Area of suitability zone of *C.argentea* in different periods (×10^4^ km^2^).

Period		Low Suit	Change%	Middle Suit	Change%	High Suit	Change (%)	Total Suit	Change%
Current		60.36	0	144.68	0	23.13	0	167.81	0
SSP126	2050s	112.85	86.96	114.72	−20.71	47.55	105.60	162.27	−3.30
	2070s	119.28	97.61	97.74	−32.44 ↓	22.56	−2.47	120.30	−28.31
	2090s	63.91	5.88	123.18	−14.86	88.91	284.40 ↑	212.09	26.39
SSP245	2050s	136.16	125.57	118.40	−18.17	25.34	9.55	143.74	−14.35
	2070s	134.66	123.10	114.87	−20.60	44.22	91.20	159.10	−5.19
	2090s	128.23	112.43	113.94	−21.25	59.86	158.78	173.79	3.57
SSP585	2050s	147.404	144.20	114.584	−20.80	29.32	26.76	143.90	−14.25
	2070s	152.160	152.08	108.038	−25.33	44.41	91.98	152.44	−9.16
	2090s	68.690	13.80	126.611	−12.49	74.49	222.07	201.11	19.84

Note: “Change (%)” represents the percentage change in suitability area compared to the current period, “↑” indicates an increase (maximal growth), “↓” indicates a decrease (maximal reduction).

**Table 6:** Area of suitability zone of *A.palmeri* in different periods (×10^4^ km^2^).

Period		Low Suit	Change%	Middle Suit	Change%	High Suit	Change (%)	Total Suit	Change%
Current		152.06	0	74.56	0	23.10	0	97.65	0
SSP126	2050s	184.57	21.38	115.63	55.09	61.61	166.77	177.25	81.50
	2070s	164.01	7.86	92.47	24.02	63.78	176.15	156.25	60.00
	2090s	146.43	−3.71 ↓	87.81	17.78	37.86	63.90	125.67	28.69
SSP245	2050s	167.83	10.37	96.23	29.07	80.26	247.51	176.49	80.73
	2070s	206.61	35.87	98.33	31.88	104.60	352.86	202.92	107.80
	2090s	193.68	27.37	108.33	45.29	108.07	367.92	216.40	121.60
SSP585	2050s	198.69	30.66	101.03	35.51	129.51	460.75 ↑	230.55	136.08
	2070s	223.35	46.88	120.59	61.73	121.22	424.83	241.80	147.61
	2090s	172.76	13.61	102.37	37.30	48.23	108.81	150.60	54.21

Note: “Change (%)” represents the percentage change in suitability area compared to the current period, “↑” indicates an increase (maximal growth), “↓” indicates a decrease (maximal reduction).

**Table 7:** Area of suitability zone of *A. spinosus* in different periods (×10^4^ km^2^).

Period		Low Suit	Change%	Middle Suit	Change%	High Suit	Change (%)	Total Suit	Change%
Current		89.64	0	91.19	0	30.83	0	122.02	0
SSP126	2050s	51.58	−42.46	104.75	14.88	92.3673	199.56	197.12	61.55
	2070s	71.32	−20.43	126.69	38.94	44.2019	43.35	170.90	40.06
	2090s	60.61	−32.38	98.62	8.16	85.3605	176.83	183.98	50.78
SSP245	2050s	73.11	−18.43	118.39	29.84	51.9556	68.5	170.35	39.61
	2070s	57.10	−36.30	104.71	14.83	93.7399	204.01	198.45	62.64
	2090s	57.09	−36.31	83.04	−8.93	127.493	313.47	210.54	72.54
SSP585	2050s	45.498	−49.24 ↓	79.617	−12.69	135.5	339.44	215.12	76.30
	2070s	56.800	−36.63	81.402	−10.73	141.982	360.46 ↑	223.38	83.07
	2090s	54.573	−39.12	99.133	8.72	90.541	193.63	189.67	55.44

Note: “Change (%)” represents the percentage change in suitability area compared to the current period, “↑” indicates an increase (maximal growth), “↓“ indicates a decrease (maximal reduction).

**Table 8:** Centroid coordinate and distance of centroid transfer of four *Amaranthaceae* plants in different periods.

Period		*D. ambrosioides*			*C. argentea*			*A. palmeri*			*A. spinosus*		
		Lon(E)	Lat(N)	Dist(km)	Lon(E)	Lat(N)	Dist(km)	Lon(E)	Lat(N)	Dist(km)	Lon(E)	Lat(N)	Dist(km)
Current		109.18	26.798	—	111.01	27.78	—	113.52	31.83	—	111.30	26.75	—
SSP126	2050s	108.58	28.858	236.44	110.42	29.99	251.64	113.88	33.74	215.36	111.48	29.00	250.52
	2070s	109.47	28.348	103.89	110.42	31.18	132.53	114.84	34.12	98.04	111.69	28.69	40.11
	2090s	109.25	27.85	59.3	111.16	28.69	284.98	115.1	33.73	48.98	111.84	28.27	47.91
SSP245	2050s	109.08	29.03	247.76	110.17	30.58	322.01	114.69	33.86	250.98	111.59	29.22	276.73
	2070s	109.42	29.26	42.6	110.36	30.22	43.91	114.57	33.99	17.91	111.74	28.98	30.59
	2090s	109.19	29.73	56.44	110.95	31.43	144.81	114.81	34.32	42.98	111.41	29.36	52.46
SSP585	2050s	109.93	29.02	258.03	110.04	30.27	292.44	114.31	31.44	264.12	111.58	29.04	256.32
	2070s	109.81	29.54	58.66	110.94	31.36	147.75	115.04	34.78	100.52	111.82	29.59	65.66
	2090s	109.3	27.52	229.69	111.03	28.67	298.79	114.93	33.96	91.73	111.81	28.30	143.29

Note: “Lon(E)” represents the longitude (Eastern Hemisphere), “Lat(N)” represents thelatitude (Northern Hemisphere), Dist(km) refers to distance (unit: kilometer); “—” indicates non-existence compared to the current context.

**Table 9:** Differences in Niche Dynamics Among four *Amaranthaceae* plants and PCA Ordination.

Species	Schoener’s D	Hellinger’s I	Stability (%)	Expansion (%)	Unfilling (%)	Equivalency (*p*)
*D. ambrosioides*	0.2691	0.4620	0.9718	0.0282	0.5407	0.0693
*C. argentea*	0.0064	0.0532	0.8955	0.1045	0.9154	0.6436
*A. palmeri*	0.5094	0.7019	0.9935	0.0065	0.5106	0.0297
*A. spinosus*	0.0266	0.051	0.027	0.973	0.973	0.07

**Table 10:** Optimal range of top contribution.

Species	Top 2 Predictor (Contribution)	Optimal Range
*D. ambrosioides*	Bio6 (44.6)	3.50–10.25 °C
	Bio12 (33.3)	1076.12–1956.18 mm
	Bio18 (9.5)	593.96–991.04 mm
*C. argentea*	Bio11 (56.4)	4.96–26.79 °C
	Bio6 (18.4)	0.14–23.10 °C
	Bio12 (7.1)	1097.36–2026.22 mm
*A. spinosus*	Elevation (27.3)	0–256.36 m
	UVB2 (16.2)	0.65–1.36
	Bio11 (15.7)	−1.11–24.91 °C
	Bio15 (12.7)	112.79–160.44%
	Bio8 (12.3)	24.42–37.49 °C
*A. spinosus*	Bio10 (25.2)	26.06–36.10 °C
	Bio7 (20.8)	8.90–27.78 °C
	Drainage (13.7)	0.6–1.02
	BSAT (12.9)	6.5–63.82%
	UVB2 (10)	0.67–12.66

## Data Availability

Occurrence data are available in GBIF [45] and CVH (http://www.cvh.ac.cn). No specific permits were required for plant occurrence data collection. The original contributions presented in the study are included in the article; further inquiries can be directed to the corresponding authors.
